# GENERATOR HEART FAILURE DataMart: An integrated framework for heart failure research

**DOI:** 10.3389/fcvm.2023.1104699

**Published:** 2023-03-22

**Authors:** Domenico D’Amario, Renzo Laborante, Agni Delvinioti, Jacopo Lenkowicz, Chiara Iacomini, Carlotta Masciocchi, Alice Luraschi, Andrea Damiani, Daniele Rodolico, Attilio Restivo, Giuseppe Ciliberti, Donato Antonio Paglianiti, Francesco Canonico, Stefano Patarnello, Alfredo Cesario, Vincenzo Valentini, Giovanni Scambia, Filippo Crea

**Affiliations:** ^1^ Department of Cardiovascular and Pulmonary Sciences, Catholic University of the Sacred Heart, Rome, Italy; ^2^ Department of Cardiovascular Sciences, Fondazione Policlinico Universitario A. Gemelli IRCCS, Rome, Italy; ^3^ Università del Piemonte Orientale, Dipartimento Medicina Translazionale, Azienda Ospedaliero-Universitaria Maggiore della Carità, Dipartimento Toraco-Cardio-Vascolare, Unità Operativa Complessa di Cardiologia 1, Novara, Italy; ^4^ Gemelli Generator, Fondazione Policlinico Universitario A. Gemelli IRCCS, Rome, Italy; ^5^ Department of Bioimaging, Radiation Oncology and Hematology, Fondazione Policlinico Universitario “A. Gemelli” IRCCS, Università Cattolica S. Cuore, Rome, Italy

**Keywords:** heart failure, big data, artificial intelligence, machine learning, datamart

## Abstract

**Background:**

Heart failure (HF) is a multifaceted clinical syndrome characterized by different etiologies, risk factors, comorbidities, and a heterogeneous clinical course. The current model, based on data from clinical trials, is limited by the biases related to a highly-selected sample in a protected environment, constraining the applicability of evidence in the real-world scenario. If properly leveraged, the enormous amount of data from real-world may have a groundbreaking impact on clinical care pathways. We present, here, the development of an HF DataMart framework for the management of clinical and research processes.

**Methods:**

Within our institution, Fondazione Policlinico Universitario A. Gemelli in Rome (Italy), a digital platform dedicated to HF patients has been envisioned (GENERATOR HF DataMart), based on two building blocks: 1. All retrospective information has been integrated into a multimodal, longitudinal data repository, providing in one single place the description of individual patients with drill-down functionalities in multiple dimensions. This functionality might allow investigators to dynamically filter subsets of patient populations characterized by demographic characteristics, biomarkers, comorbidities, and clinical events (e.g., re-hospitalization), enabling agile analyses of the outcomes by subsets of patients. 2. With respect to expected long-term health status and response to treatments, the use of the disease trajectory toolset and predictive models for the evolution of HF has been implemented. The methodological scaffolding has been constructed in respect of a set of the preferred standards recommended by the CODE-EHR framework.

**Results:**

Several examples of GENERATOR HF DataMart utilization are presented as follows: to select a specific retrospective cohort of HF patients within a particular period, along with their clinical and laboratory data, to explore multiple associations between clinical and laboratory data, as well as to identify a potential cohort for enrollment in future studies; to create a multi-parametric predictive models of early re-hospitalization after discharge; to cluster patients according to their ejection fraction (EF) variation, investigating its potential impact on hospital admissions.

**Conclusion:**

The GENERATOR HF DataMart has been developed to exploit a large amount of data from patients with HF from our institution and generate evidence from real-world data. The two components of the HF platform might provide the infrastructural basis for a combined patient support program dedicated to continuous monitoring and remote care, assisting patients, caregivers, and healthcare professionals.

## Introduction

Heart failure (HF) contributes to a significant proportion of the global burden of cardiovascular diseases, with increasing prevalence and incidence rates worldwide (1). Currently, it is estimated that approximately 2% of adults suffer from HF in industrialized countries, although the true prevalence is likely to be higher, due to the common underestimation of this clinical syndrome (2).

The prognosis of patients with HF has improved considerably in the last decades since several drugs have been developed and tested in randomized controlled trials (RCTs). However, the improvement in overall survival has been confined mainly to those with heart failure with reduced ejection fraction (HFrEF) and the quality of life (QoL) remained poor in the advanced phases of the disease (1). Furthermore, acute HF continues to represent one of the greatest unmet needs in cardiovascular medicine, as trials of novel interventions have been largely unsuccessful (1, 3). In addition, RCTs in HF are becoming increasingly elaborated, expensive, time-consuming, and limited to a selected population, excluding broad categories of patients, such as those with chronic kidney disease or hyperkaliemia (4). In this regard, Real World Data (RWD), derived from several sources, including electronic health records (EHRs) (i.e., systematized collection of patient's health information stored in a digital format) and registries, may provide extensive and generalizable data from the real-world ground, enabling the validation of risk markers, risk scores, and drug usability. As Dr. Lukas Kappenberger, a pioneer in computational cardiology, said in 2005, “Science (i.e., RCTs) tells us what we can do; guidelines what we should do; and registries what we are doing”(5). However, RWD-based studies require long-term data collection, high set-up, and running costs, usually including a specific set of predefined variables captured at specific time points and periods (5). Therefore, the significant scale, complexity, and speed at which such data are collected necessarily require innovative approaches that exploit the use of automated data investigation and discovery (6). Through the automated process of artificial intelligence (AI) and machine learning, the extraction and analysis of RWD could even be performed without needing to be explicitly programmed, due to a machine's ability to learn and efficiently achieve complex tasks autonomously (6). Moving towards a more data-driven paradigm, capturing data from multiple data sources and in a longitudinal manner continuously over time, our institution, Fondazione Policlinico Universitario A. Gemelli in Rome (Italy) has established a dedicated research facility “Gemelli Generator Real World Data”. The main purpose of this framework was to generate “Real World Evidence” (RWE) from RWD (i.e., observational data obtained outside the context of RCTs during routine clinical practice) related to patients with various diseases admitted to our institution (7). The facility is made up of a multidisciplinary team, composed of data scientists, medical researchers, and service design experts, cooperating to develop and deploy data-driven techniques (from data integration to data visualization and AI-based predictive models) in a standardized and clinically-validated manner. Currently, the laboratory has built a solid and reproducible methodology, usable for various medical domains, such as breast, ovarian, and colon-rectal cancers (7, 8) (Supplementary material, section “Gemelli GENERATOR DataMart framework”). In this context, GENERATOR HF DataMart was created to leverage hospitals' large amounts of data on patients with HF, generating RWE in this area, and eventually modifying the risk stratification and the clinical standard of care, adapting to contemporary rapid advances in precision medicine.

The primary aim of this work is to show, in detail, the methodology and the technologies adopted within “GENERATOR HF DataMart”, integrating a daily-updated DataMart, a data visualization dashboard, and an AI-based toolset, applied to the domain of HF.

Additionally, we provide concrete examples of how GENERATOR HF DataMart can be used to support research and clinical practice, as well as the promise, pitfalls, near-term challenges, and opportunities for big data in the HF-associated research field.

## Methods

### GENERATOR HF DataMart analytics framework

**Figure 1:**
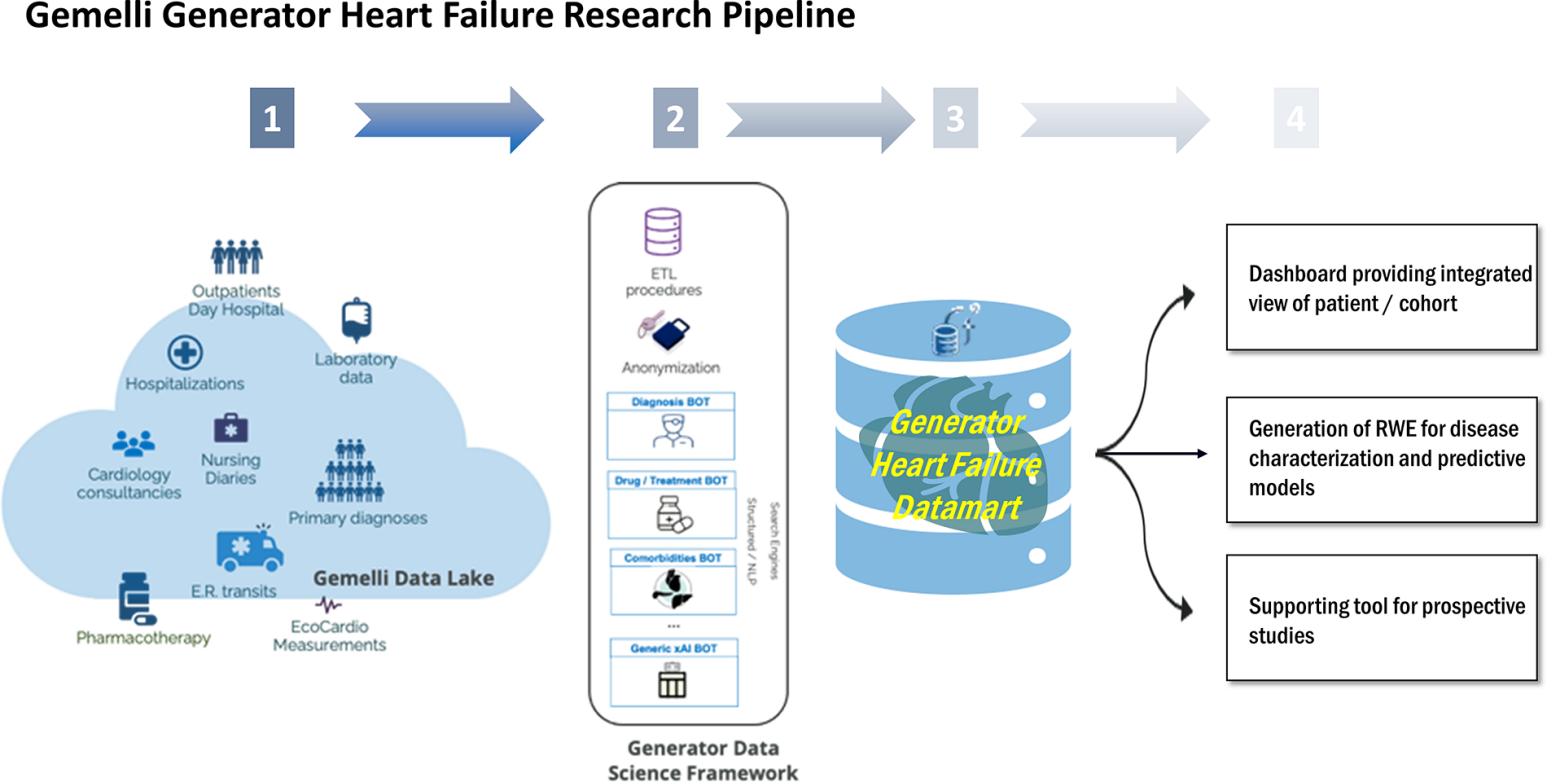
Overview of HF daily-updated analysis framework. ETL, Extract, Transform, Load; ER, Emergency Room; NLP, Neural Language Processing; RWE, Real World Evidence; xAI, eXplainable Artificial Intelligence.

A DataMart is a curated data repository that includes a subset of data from the hospital's information technology (IT) warehouse, about a specific domain or subject and allowing to analyze with integrated views of patients' clinical histories, outcomes, and biomarkers. Specifically, as depicted in Figure 1, the GENERATOR HF DataMart aims to make all relevant information residing in Gemelli Data Lake exploitable for analytics and clinical studies in the HF domain. The technological tools used are SAS^(R)^, R, and Python, which are among the most common programming environments in data science. SAS^(R)^ is used as a middleware for Extract Transform and Load (ETL) tasks from hospital information systems, as a data repository including tables in dedicated storage areas (SAS VIYA *Caslibs)*, as a text mining tool for extracting clinical concepts (such as comorbidities), and as a data visualization tool. R and Python are used for data analytics, data processing, and modeling activities.

The framework, stemming from GENERATOR infrastructure and data science system, includes three distinct components; a dynamically daily-updated datamart, a data visualization dashboard, and a set of AI modeling tools, which collective constitute GENERATOR HF DataMart

Specifically:

1) Data concern patients hospitalized for the first time and with a primary diagnosis of HF. The DataMart captures daily updated clinical, laboratory, and instrumental data, related to each contact, defined as a patient's access, either on an outpatient or inpatient basis, differentiated, admitted in the elective, ambulatory, or emergency ward. In particular, the DataMart is implemented in a dedicated SAS^(R)^ VIYA Caslib where the data is transformed, extracted, and loaded from the hospital information systems;
2) The data visualization dashboard through which patient data can be filtered dynamically according to specific selection criteria, such as demographics, biomarkers, or comorbidities. The data visualization dashboard is implemented in SAS^(R)^ VIYA environment and Visual Analytics;
3) The set of AI-based modeling tools, leveraging the up-to-date industry standards in terms of statistical analysis and machine learning, using cutting-edge algorithmic techniques, based on open-source packages in R and Python. Training and validation sets including subsets of the complete data list are specified per the study of interest and are available in SAS^(R)^ VIYA Caslibs and accessible with the *swat* library from Python and R notebooks, running either locally or in dedicated virtual machines.

Of note, according to the CODE-EHR framework, our methodology, explained in detail throughout this manuscript, overall meets the preferred standards in all the items (9). In particular, the *dataset construction and linkage* item is addressed in the methods section “Data sources and evidence generation workflow” and in the Supplementary material section “Overview of data extraction procedures”. Regarding the *data fit for purpose* item, we have detailed the complete Data Ontology considered in GENERATOR HF Datamart (Supplementary Table S2). Furthermore, as far as the *disease and outcome definitions* and *analysis* items, we provided outcome definitions, together with the corresponding modeling use cases, in the results section “Re-hospitalization modeling and patient clustering as a function of EF trend”. Furthermore, the coding procedures per variable are discussed in Supplementary material, section “Data extraction workflow”. Diagnoses are derived from the ICD-9 codes, whereas diseases and comorbidities are assigned according to the latest European Society of Cardiology (ESC) guidelines and further confirmed and validated following the Joint Commission International standards since Fondazione Policlinico Universitario A. Gemelli is an accredited institution. Finally, all privacy issues were analyzed jointly with Policlinico Gemelli Data Protection Officer, to design an approach fully compliant with the Italian and European GDPR directives and regulations (EU Directive 2016/679 and under Italian Laws: Decreto Legislativo 196/2003, Decreto Legislativo 101 2018, Autorizzazione Generale Garante 9/2016). These principles of Ethics and Governance are clearly stated in a legally relevant public document, the Generator Real World Data Facility Umbrella Protocol, on which further details can be given upon reasonable request. For more details, please refer to the Supplementary material section Gemelli Generator Real World Data ethics and governance.

### Automated analytics for GENERATOR HF DataMart build

**Figure 2:**
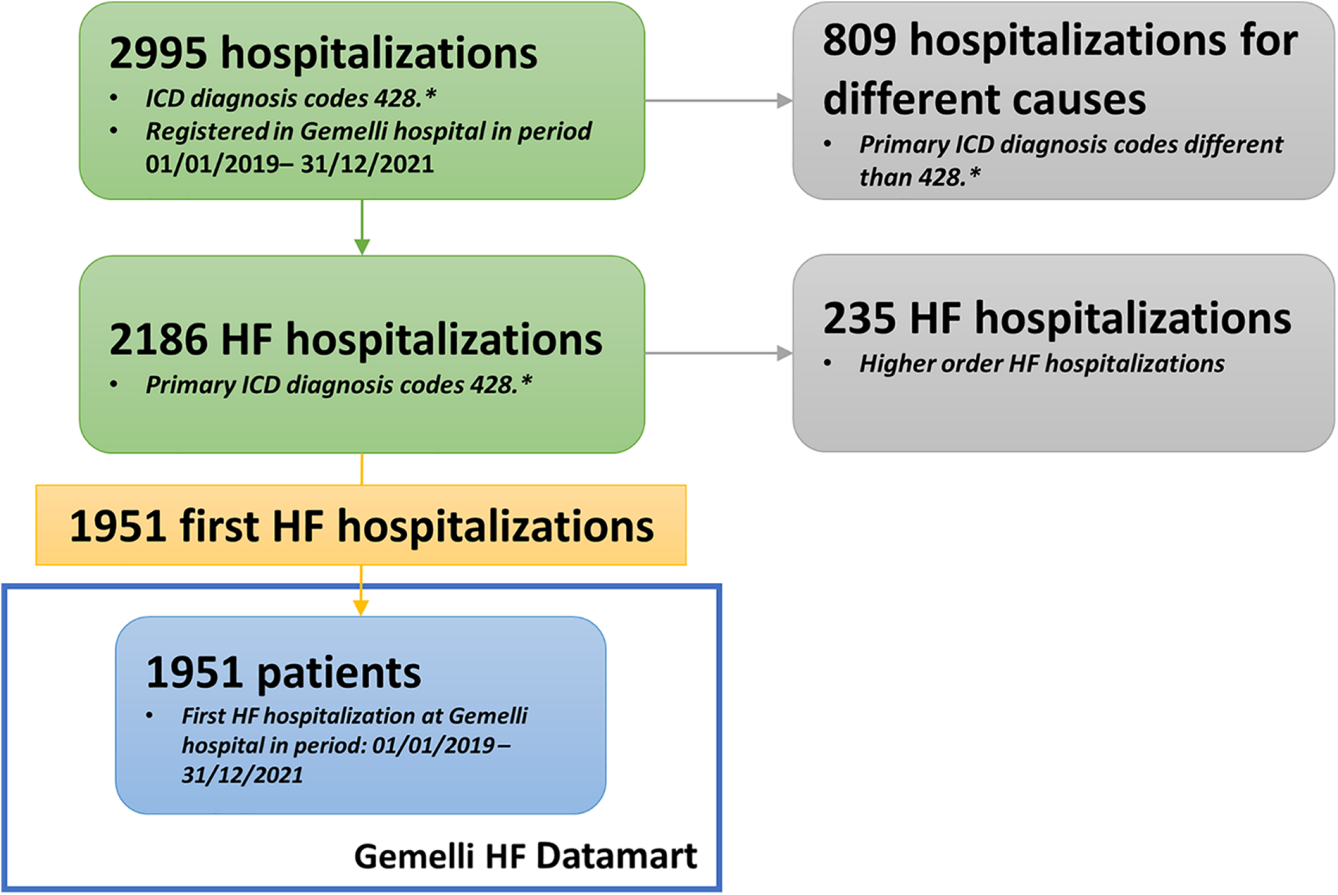
Data mart creation methodology. HF, Heart Failure; ICD, International Classification of Disease.

Every domain-specific-GENERATOR DataMart framework is the result of cooperation and knowledge exchange between clinicians and specialists of the domain under study, data scientists, and data analysts. The general workflow for setting up a DataMart is shown in Figure 2. First, clinicians indicate the inclusion criteria (mainly based on the codes from the International Classification of Disease 9, ICD-9 428, recorded in the hospital discharge diagnoses) and a primary list of variables of interest, including biomarkers, demographics, functional data, comorbidities, therapies, and outcomes. Based on the specific ICD-9 428 diagnosis codes, data scientists and analysts select from health records patients matching the inclusion criteria and then perform the mapping of all variables into the hospital data sources and subsystems. This repeatable sequence of activities constitutes the systematic approach to GENERATOR DataMart implementation, regarding data collection and standardization.

Iterative design sessions are regularly performed to meet clinicians’ demands and develop a user-oriented data visualization dashboard.

Regarding the specific GENERATOR HF DataMart framework, a dedicated working group has been formed with the task of identifying a variable set of interest, the so-called HF data ontology, and specific inclusion criteria [i.e., patients who have been hospitalized with a primary diagnosis of HF (ICD-9 codes 428.* at discharge) during 2019–2021 period] (Supplementary Table S1, Table S2, Figure S2).

The technological components used to develop the GENERATOR HF DataMart framework are part of the standard toolset that the GENERATOR center has developed and progressively improved through the constant interaction with medical teams across the different medical areas.

These are represented by:

1) SAS® Institute suite for data extraction, transformation, and load, to ensure smooth interoperability and integration between hospital informative technology (IT) systems and data warehouse;
2) R-Studio and/or open-source suites, to analyze medical notes and identify information of interest (such as comorbidities, risk factors, etc.);
3) SAS^(R)^ VIYA for data visualization dashboard;
4) R^(R)^ and Python^(R)^ open-source libraries for the prototyping and implementation of statistical analysis and machine learning.

Interoperability represents a key point in data management for properly exploiting their resources, for example, to visualize them for different stakeholders, or through joint statistical analysis at the European level (10). Our approach to interoperability is twofold, as shown in another area of application (11). First, we standardize diagnoses and treatments, according to ICD-9 428 and Anatomical Therapeutic Chemical (ATC) classification systems, to adapt GENERATOR HF DataMart to the FHIR (Fast Healthcare Interoperability Resources) specification (12). Second, portability to other hospitals, at the Italian and European levels, can be achieved since we apply FAIR (Findability, Accessibility, Interoperability, and Reuse of digital assets) principles to data, metadata, and infrastructure (13). Furthermore, we leverage federated data processing methods [i.e., distributed learning algorithms (14)], which allow us to establish interoperability and collaboration among hospitals, using the same statistical and machine learning methods, without transferring data from these hospitals to a centralized site, to be compliant with the General Data Protection Regulation [GDPR, Regulation (EU) 2016/679].

### Data sources and evidence generation workflow

The GENERATOR HF DataMart integrates heterogeneous data from multiple sources in form of structured or unstructured data. Structured data is characterized by a high level of standardization and fulfill certain criteria, in terms of ontologies and data formats (data model) (for example, ICD-9 428 code for diagnosis). Such data is entered without further processing into GENERATOR HF DataMart in dedicated tables, grouped by data source and/or data category, establishing “relational databases”. Conversely, unstructured data refer to unorganized data, such as medical records in plain text, which require further processing before being integrated into our GENERATOR HF DataMart.

Natural language processing (NLP) and text mining techniques allow for the extraction of clinically relevant variables from plain text and their integration in the GENERATOR HF DataMart. NLP and text mining are performed using either SAS^(R)^ VIYA Text Analytics for concept extraction (i.e., comorbidities, risk factors) or native Python libraries such as *re* for the use of regular expressions. Regular expressions identify predefined keywords of interest; while distance-based rules filter negations and expressions referring to familiarity. More advanced NLP use cases are explored for topic modeling and classification of clinical reports using both traditional features and word frequencies (package *scikit-learn* and *gensim*) and sentence embedding (package *sentenceTransformers*).

Therefore, data extraction is performed based on ETL procedures, differentiated per data type (structured and unstructured) (Supplementary Figure S1). As the final step in the data extraction workflow, extensive validation procedures ensure the consistency and the quality of their transformation. Data validation is performed in different phases of data extraction and transformation including standardized reporting processes. Regarding structured and calculated variables, data analysts and data scientists examine variable distributions and their acceptable values before consolidating the respective ETLs. Concerning unstructured data and text, extracted variables are initially validated by the technical team and later independently by clinicians using dedicated annotation tools (open source such as *doccano* or made in-house). Evaluation reports on extracted concepts assist to iteratively define and optimize text mining rules. Finally, regarding variables presenting a direct relationship between them (i.e., BMI and obesity), we examine whether the relationship conditions are met as an index of data consistency. A detailed overview of the entire data extraction workflow is provided in the Supplementary material, section on data quality and completeness.

An extensive list of clinical data was selected and captured in the GENERATOR HF DataMart, fulfilling the ambition to build the most comprehensive and longitudinal overview of HF patients within our institution. Supplementary Table S2 shows the complete overview of the HF data ontology. The main categories of structured data are demographics, admissions, contacts, laboratory exams, echocardiograms, ECG, and outcomes (death, re-hospitalization, and admissions for acute events in the emergency department). Instead, the most relevant unstructured data are represented by comorbidities, risk factors, medications, interventions, and procedures.

The HF framework is regularly updated with new data and new patients, as follows:

1) data related to patients, already included in the cohort, are daily updated when a new contact occurs;
2) once a new patient meets the predefined inclusion criteria (i.e. admission for the first time at our institution with a primary diagnosis of HF), he/she is inserted in the patient cohort. Subsequently, his/her data is entered in the GENERATOR HF DataMart based on the data workflow, previously described.

## Results

In this section, we provide some examples of how GENERATOR HF DataMart has been exploited. A detailed description of the results is beyond the scope of this manuscript, the purpose of which is primarily to provide a description of the data collection and processing methodology. The results shown are only a hint of how our DataMart could be implemented in clinical practice and research.

### Retrospective cohort selection

**Figure 3:**
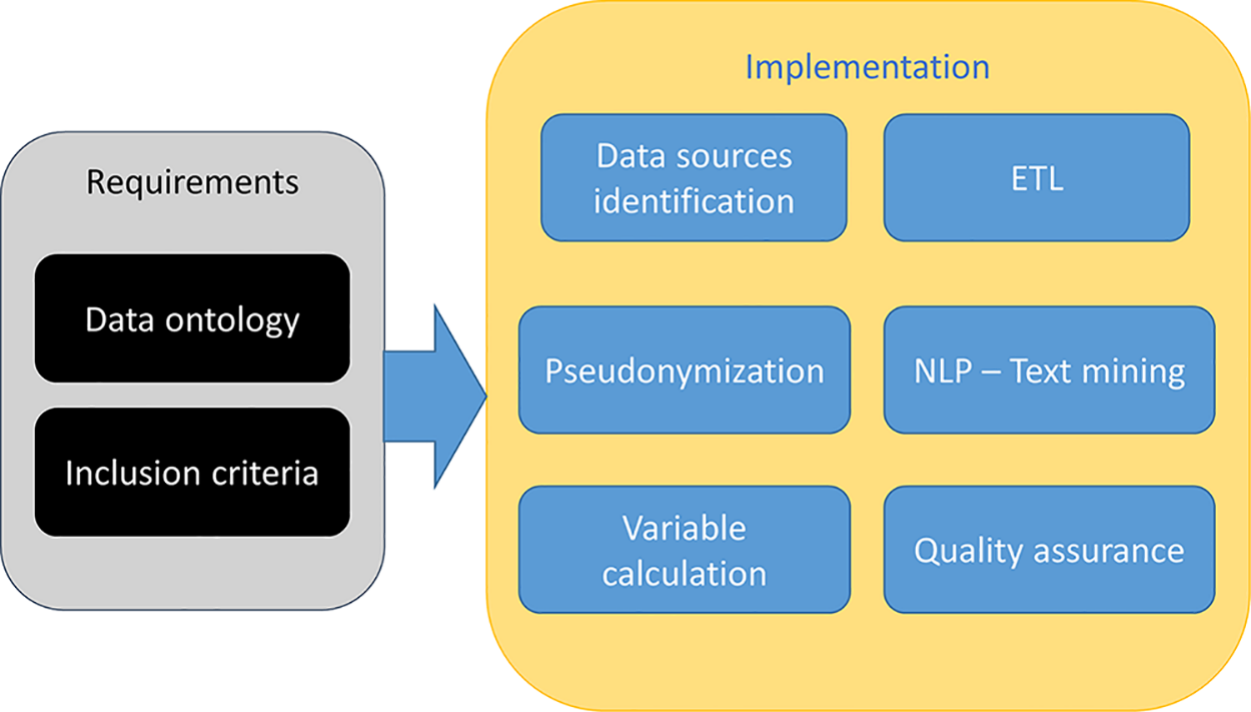
Flowchart describing cohort selection. ETL, Extract, Transform, Load; NLP, Neural Language Processing.

Considering 2019–2021 as the reference period, we selected the group of patients who have been hospitalized with a primary diagnosis of HF (ICD-9 codes 428.* at discharge), with the possibility to validate it with the values of instrumental examinations, such as an echocardiogram, biomarkers (i.e., nt-proBNP) and clinical data, to render our inclusion criteria more stringent, but at the same time more confident (Figure 3). The final cohort included 1951 patients. Such a selection provides a comprehensive overview of HF patients currently under treatment at our institution, together with their clinical and laboratory data.

Table 1 shows an overview of the main clinical characteristics, biomarker values, and outcomes at the discharge of the first hospitalization for HF in this subset of patients. Continuous variables are reported as the median and interquartile range (IQR), and categorical variables are as counts and proportions (%). The median age of the overall population was 78 years (IQR 68–85) and 58.3% were male. Among 829 patients with an available measurement of ejection fraction (EF) before re-hospitalization, 318 patients had HFrEF, 160 patients had Heart Failure with mid-range Ejection Fraction (HFmrEF) and 351 patients had Heart Failure with preserved Ejection Fraction (HFpEF), according to the most recent ESC definition (1). 66.3% presented hypertension, 30.2% diabetes, and 28.4% a pulmonary disease. As regards clinical outcomes, 5.4% died during hospitalization while 8% of patients have been readmitted within 30 days after discharge. Table 2 provides an overview of the number of contacts, per year, and contact type. Contact number is reported as a median value among patients, together with its interquartile range (IQR) per contact type (outpatient, day hospital, in-patient, emergency) and per year.

**Table 1:** Baseline characteristics at hospitalization discharge for retrospective cohort 2019–2021.

Variables	Total patients (*n* = 1951)	Missing rate
Demographics/organizational/socioeconomic		
Age (years), median(IQR)	78.0 (68.0,85.0)	0 (0.0%)
Male sex	1,138 (58.3%)	0 (0.0%)
Education		648 (33.2%)
No education	51 (2.6%)	
Primary	318 (16.3%)	
Secondary	746 (38.2%)	
Higher	188 (9.6%)	
Married	969 (49.7%)	1 (0.1%)
Clinical		
EF Class		1,122 (57.5%)
HFrEF	318 (16.3%)	
HFmrEF	160 (8.2%)	
HFpEF	351 (18.0%)	
NYHA		1,441 (73.9%)
I	42 (2.2%)	
II	56 (2.9%)	
III	322 (16.5%)	
IV	90 (4.6%)	
Heart rate (bpm), median (IQR)	74.0 (66.5,80.0)	667 (34.2%)
Systolic blood pressure (mmHg), median (IQR)	120.0 (110.0,130.0)	656 (33.6%)
BMI(kg/m^2^), median (IQR)	26.0 (23.4,29.4)	621 (31.8%)
Laboratory values		
Hemoglobin(g/dl), median (IQR)	12.2 (10.5,13.7)	10 (0.5%)
NT-ProBNP(pg/ml), median (IQR)	3492.5 (1280.2,8815.0)	265 (13.6%)
eGFR (ml/min/1.73 m^2^), median (IQR)	60.9 (40.2,84.0)	10 (0.5%)
Potassium (mEq/L), median (IQR)	4.0 (3.7,4.4)	9 (0.5%)
History and comorbidities		
Diabetes	589 (30.2%)	0 (0.0%)
Pulmonary disease	554 (28.4%)	0 (0.0%)
Malignant disease	416 (21.3%)	0 (0.0%)
Hypertension	1,293 (66.3%)	0 (0.0%)
Hepatic disease	34 (1.7%)	0 (0.0%)
Treatment		
β-blockers	1,410 (72.3%)	0 (0.0%)
ACEi	377 (19.3%)	0 (0.0%)
ARB	171 (8.8%)	0 (0.0%)
ARNi	887 (45.5%)	0 (0.0%)
MRA	564 (28.9%)	0 (0.0%)
SGLT2i	49 (2.5%)	0 (0.0%)
Diuretics	1,460 (74.8%)	0 (0.0%)
Digoxin	117 (6.0%)	0 (0.0%)
Statin	786 (40.3%)	0 (0.0%)
Acetylsalicylic acid (ASA)	324 (16.6%)	0 (0.0%)
Outcomes		
Re-hospitalization within 30 days	156 (8.0%)	0 (0.0%)
In hospital death	106 (5.4%)	0 (0.0%)

All included variables reported at the date of discharge.

ACEi, angiotensin-converting enzyme inhibitor; ARB, angiotensin receptor blocker; ARNi, angiotensin receptor–neprilysin inhibitor; BMI, body mass index; eGFR, estimated glomerular filtration rate; IQR,interquartile range; MRA,mineralo corticoid receptor antagonist; NYHA,New York Heart Association;NT-proBNP, N-terminal pro hormone brain natriuretic peptide; SGLT2i,Sodium-glucose co-transporter 2; EF Class, Ejection Fraction Class; HFrEF. Heart Failure with Reduced Ejection Fraction; HFmrEF, Heart Failure with Mid-Range Ejection Fraction; HFpEF, Heart Failure with Preserved Ejection Fraction.

**Table 2:** Contact statistics per year and type of contact for the retrospective cohort 2019–2021.

Year	Contact Type	Number of contacts [median (IQR)]
2019	Outpatient	2.0 (1.0,4.0)
	Day Hospital	1.0 (1.0,2.0)
	Emergency	1.0 (1.0,2.0)
	Inpatient	1.0 (1.0,2.0)
2020	Outpatient	2.0 (1.0,3.0)
	Day Hospital	1.0 (1.0,2.0)
	Emergency	1.0 (1.0,2.0)
	Inpatient	1.0 (1.0,2.0)
2021	Outpatient	2.0 (1.0,3.0)
	Day Hospital	1.0 (1.0,1.5)
	Emergency	1.0 (1.0,2.0)
	Inpatient	1.0 (1.0,2.0)

This cohort could be exploited to investigate multiple associations between clinical and laboratory data, as well as represent a contemporary cohort for enrollment in future studies.

### Data visualization to support clinical study design

**Figure 4:**
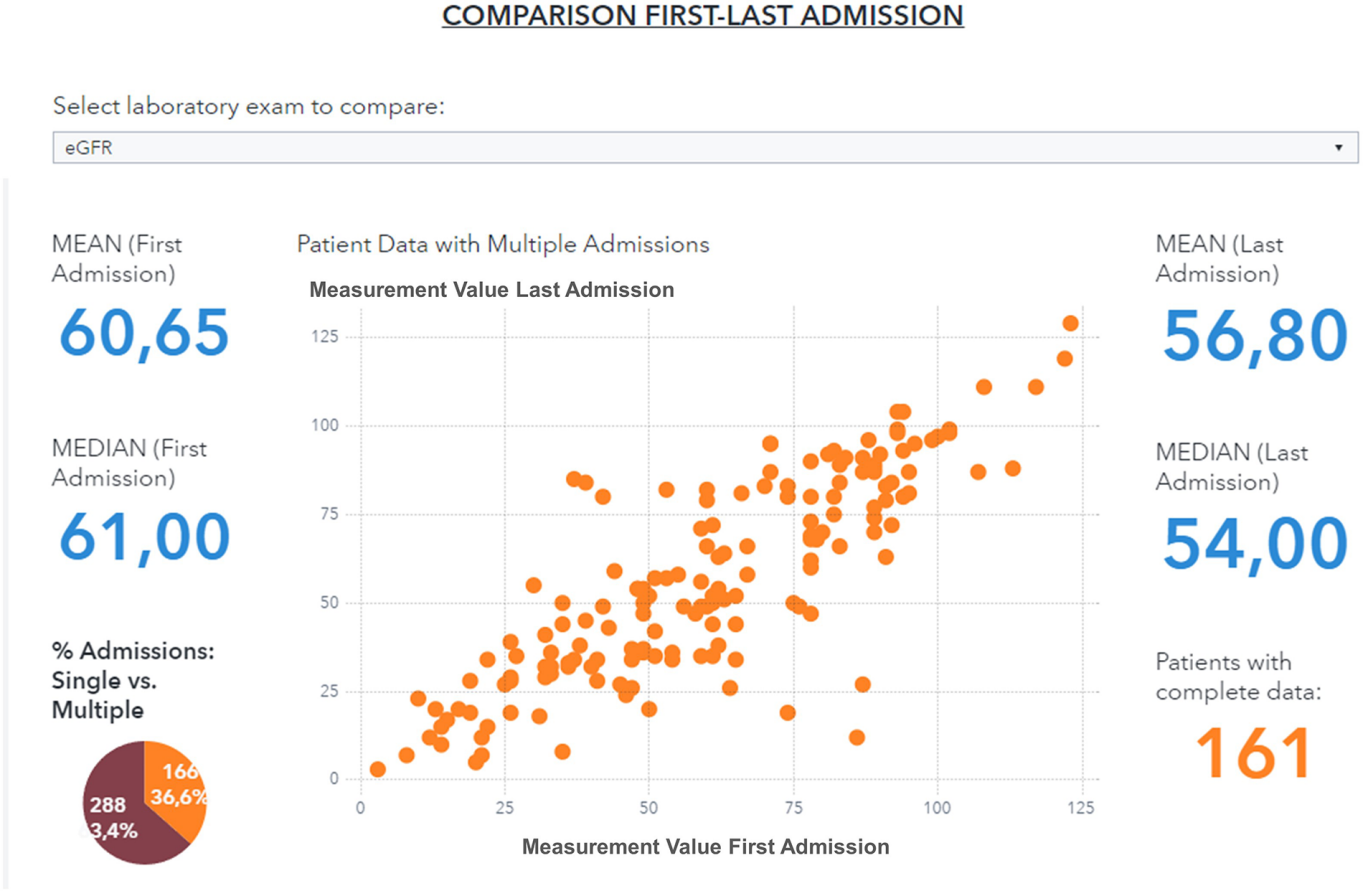
Data visualization of selected variables (eGFR, Hemoglobin, NT-ProBNP) distribution for a patient subgroup during the first hospitalization event (patients with age <90 years, diabetes, and hypertension, period 2019–2021). eGFR, estimated Glomerular Filtration Rate.

**Figure 5:**
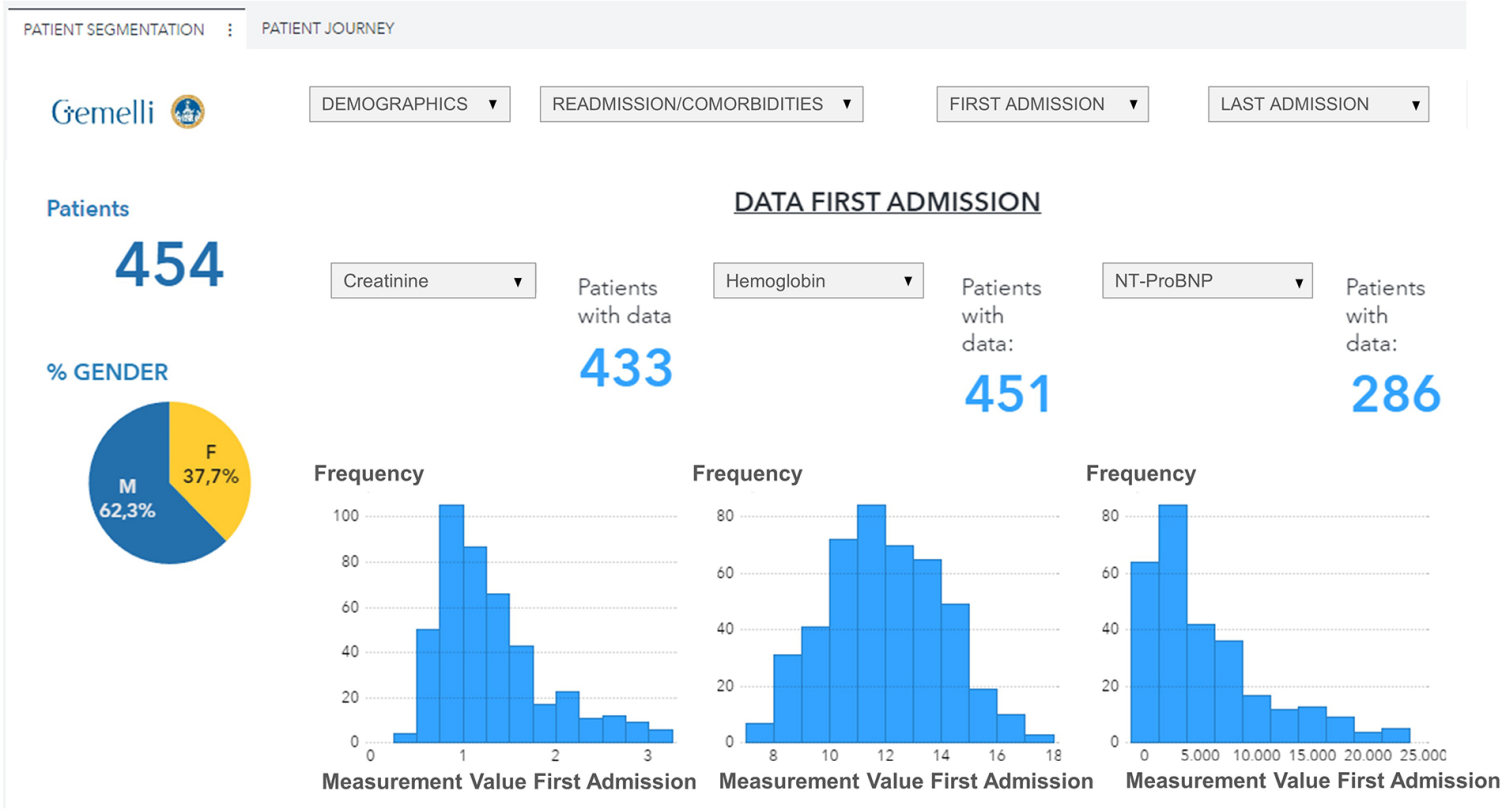
Comparison of eGFR values between first and last hospitalization event for a patient subgroup (patients with age <90 years, diabetes and hypertension, period 2019–2021).

The data visualization component of the HF framework offers two complementary views, cohort segmentations, and patient journeys. Once a cohort is identified, several types of data groups can be selected for analysis, as shown in Figure 4. The user can select subgroups with common features (i.e., age, specific comorbidities, re-hospitalization events) and analyze the distribution of biomarkers for this subgroup (“cohort segmentation” utility) at discharge. This utility may be used as a tool to compose cohorts with specific features that can be later screened for participation in clinical trials. In the same view, it is possible to compare selected features such as estimated glomerular filtration rate (eGFR) and/or hemoglobin, between the first hospitalization and the last one (Figure 5). This functionality allows clinicians to follow laboratory trends, helping them to identify some biomarkers to consider potentially during study design.

**Figure 6:**
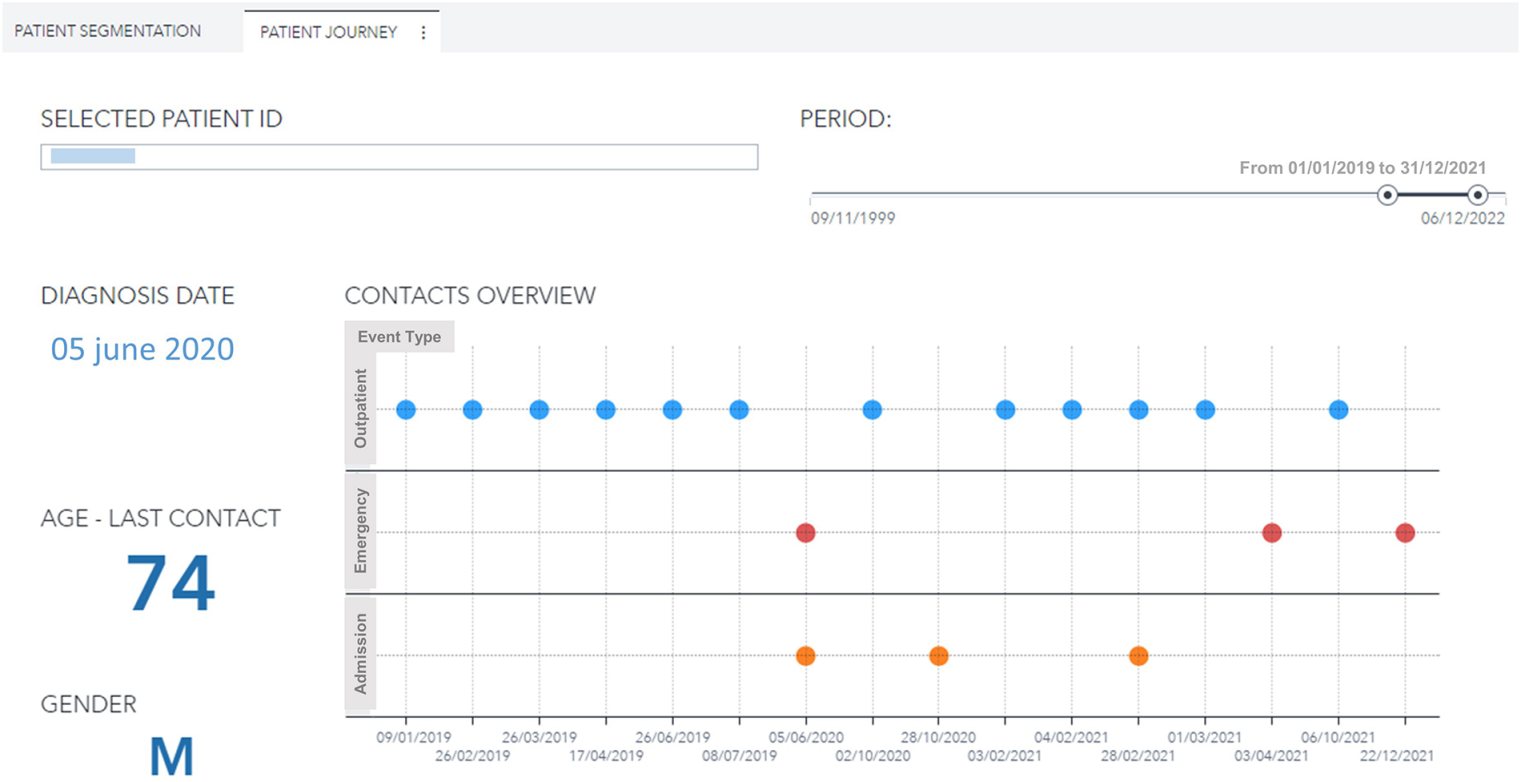
Patient journey visualization with history of contacts (outpatient, emergency, day hospital, inpatient) for a male, 74 years old patient with diabetes and hypertension during the period 2019–2021. ID, identification.

**Figure 7:**
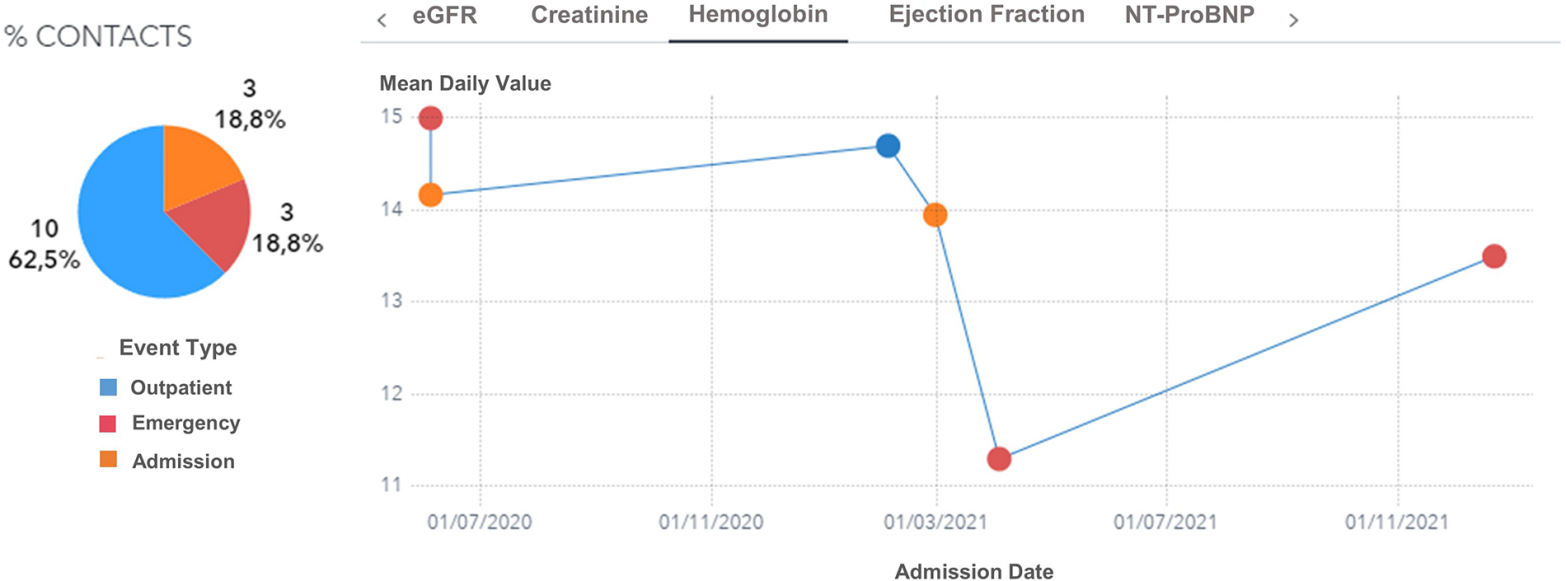
Hemoglobin trend across contacts (outpatient, emergency, day hospital, inpatient) for a male, 74 years old patient with diabetes and hypertension during the period 2019–2021. eGFR: estimated Glomerular Filtration Rate.

Moreover, the user can further drill down, select a specific patient, and visualize his complete clinical history (Figures 6, 7). For every patient included in the GENERATOR HF DataMart, it is possible to get a comprehensive view of all his contacts together with respective clinical and laboratory variables (“patient journey” utility).

### Re-hospitalization modeling and patient clustering as a function of EF trend

We focused on two distinct outcomes: re-hospitalization events within certain periods defined as the second hospitalization event observed after patient inclusion in the study and EF changes over time considering the first and last available EF measures after patient inclusion, with the aim of predicting the evolution of the disease.

Re-hospitalization modeling may allow the identification of predictors related to re-hospitalization events within 30, 60, or 90 days after discharge, employing time-to-event analysis. After extensive experimentation, we identified models with the highest true positive rate, given a certain threshold of false positive rate. Re-hospitalization events were modeled through Cox models and cross-validation techniques. Model performance was evaluated at selected time points using standard classification evaluation metrics and ROC curves in which the rate of true positives was optimized based on Jouden's statistic. Successively, we selected a list of possible predictors to compose a score that classifies patients according to the risk of re-hospitalization (i.e., high, medium, or low risk). Such scores can be integrated into the data visualization framework and can further assist clinicians during patient selection for clinical trials.

As regards patient clustering, we analyzed EF changes with the k-means algorithm on standardized variables. Considering baseline phenotype (HFrEF, HFmrEF, and HFpEF), we clustered patients based on their EF variation between the first and the last measurement and, subsequently, investigated its impact on hospital accesses. Additionally, non-linear models of EF decrease per baseline EF group may identify important predictors of EF deterioration, helping with disease management and allocation of resources. Furthermore, we may identify patterns of fast EF deterioration across different baseline EF groups and compare them to the expected trajectories in terms of hospitalizations, days in hospitals, and planned medical examinations.

## Discussion

In the current work, the authors aim to describe, in detail, the methodological scaffolding underlying GENERATOR HF DataMart, an AI-based laboratory based on RWD about patients with HF in Fondazione Policlinico Universitario A. Gemelli. This has led to the creation of a flexible, highly reusable framework for the generation of RWE, which is now being exploited for multiple research threads in the domain of HF, including the personalization of medical interventions. Therefore, the results reported in this paper are intended only as illustrative examples of such exploitation patterns, which are to be adequately described in subsequent works.

Due to the latest technological developments, cardiovascular research is stepping into an unprecedented new era, characterized by the generation and release of an incredible amount of data, termed big data (15).

Key features of them are:

1) speed, as they can be generated, processed, and analyzed in real-time;
2) quantity, due to the richness of the data, the scale of which challenges classical storage, processing, and analysis approaches;
3) variety, referring to the diversity of data sources (i.e. administrative, patient-reported, or healthcare-generated)
4) veracity, concerning their quality and reliability;
5) value, about their applicability and usability (15).

By definition, big data cannot be analyzed through traditional methods, but their management must necessarily rely on AI, defined as a set of programs that allow machines to mimic human behaviour (16). This has placed researchers in front of new challenges including data integration, transformation, verification, validation, and data privacy, which need to be necessarily resolved and explained thoroughly before presenting results. To ensure their clarity and transparency, we have described, in advance, the methodology of our project, aiming at transforming RWD into RWE in the HF field. Unquestionably, one of the major pitfalls of “traditional” clinical trials, conducted with specific populations and in specialized environments, is the lack of generalizability (17). They rely upon long lists of eligibility criteria, detailed case reporting forms that exist separately from standard medical records, accurate monitoring, and specialized research staff to ensure adherence to a well-characterized protocol (18). This allows for high-quality and accurate data, with high internal validity, but often comes at the expense of generalizability. Furthermore, “traditional” clinical trials may provide scarce data on interactions with concomitant diseases and treatments, and adherence to the tested therapy may be biased by being supported through intensive efforts, not feasible in real clinical practice (18). It should be considered that generalizability is inherently constrained because our DataMart consists of a geographically-limited patient cohort. Therefore, an essential point for maximizing generalizability is to ensure interoperability with other “datamarts” from other research contexts. This is ensured since each variable is standardized and defined according to the latest European guidelines, enabling the reproducibility of our analysis in other environments.

Acute and chronic HF may represent a promising target for big data use, due to its intrinsic complexity and heterogeneity. Clinical big data, collected during daily clinical practice, reflect real-life populations, providing a complementary viewpoint compared to rigorous and highly selective RCTs. They may be extracted from imaging exams, EHRs, and implantable or wearable devices. However, RWD, despite increased generalizability, raises other concerns about data quality and data missingness, as a downside. Indeed, they are not collected to support a specific research project defined *a priori*. Therefore, their accuracy and reliability need to be taken into account and maximized, as far as possible, for such purposes.

The fields, within which GENERATOR HF DataMart could be employed, are multifarious.

First, HF 30-day readmission rates have been a major focus of efforts to improve the prognosis and QoL of HF patients, as well as reduce healthcare costs (1). Unlike other diseases like acute coronary syndrome and pneumonia, which are most often isolated events not expected to imminently recur, acute HF patients are at increased risk for early re-admission since they have higher rates of comorbidities or residual congestion at the time of discharge (19). Different risk scoring systems have been designed to identify patients at high risk of 30-day re-admission in the general population, but none of these predictive risk scores has been widely implemented in the population of HF (20). Nevertheless, the identification of this subgroup of patients at high risk of re-admission could allocate appropriate resources to these patients, as well as attenuate the clinical and financial burdens of overall patients.

Second, according to contemporary guidelines, HF classification and treatment decisions are deeply based on the evaluation of EF (1). However, EF is not a stationary parameter but can increase or decrease over time. Of note, the majority of contemporary studies focus exclusively on baseline EF and its recovery, without assessing the full spectrum of EF changes, its determinants, and related prognosis (21–23). As a result, information on the onset, determinants, and prognosis of EF variation over time is scant and limited to a handful of studies (24–27). The main reason for this knowledge gap is the lack of granularity of clinical trial data, often with a short follow-up and a narrow data spectrum, specific to certain research purposes. On the other hand, GENERATOR HF DataMart, including demographics, vitals, diagnosis, labs, procedures, medications, and their response, represents potentially a patient's multi-parametric health trajectory, capable of filling this gap.

**Figure 8:**
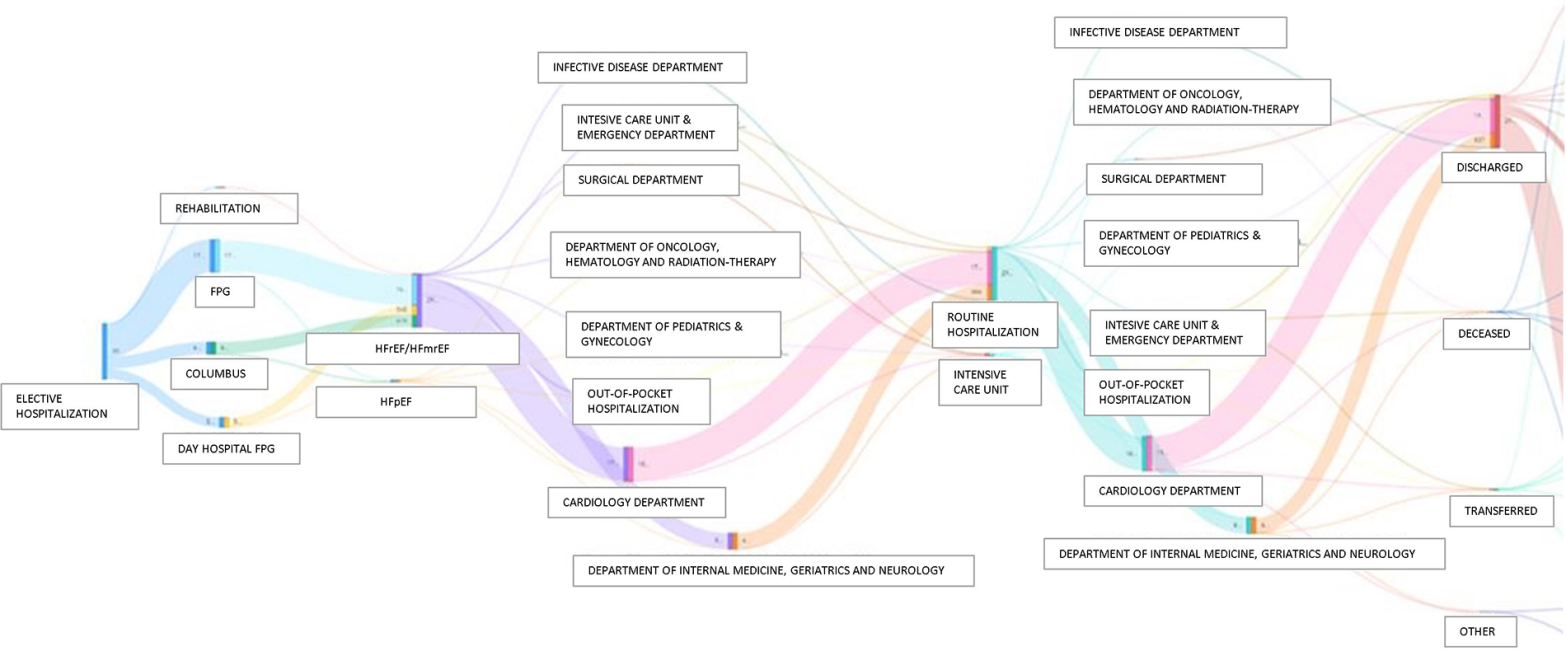
Sankey diagrams grouping HF patients according to their clinical pathways within Policlinco Gemelli between 2019 and 2021. FPG, Fondazione Policlinico Gemelli; HFpEF, Heart Failure with preserved Ejection Fraction; HFrEF, Heart Failure with reduced Ejection Fraction; HFmrEF, Heart Failure with mildy reduced Ejection Fraction.

Third, one of the greatest challenges in modern cardiology is “precision medicine,” which means delivering therapies tailored to each patient, taking inter-individual variability into utmost consideration. However, precision medicine requires more detailed data, together with a considerable ability of computers to analyse, integrate and leverage these data, to create the “digital twin” of a patient (28). In healthcare, it represents a comprehensive and virtual tool that integrates coherently and dynamically the clinical data acquired over time for an individual and creates a digital model of him, projected into the future. In practice, a “digital twin” may suggest whether a treatment is appropriate for a patient by simulating drug response before a specific treatment is ultimately chosen (28). One emerging application of the “digital twin” is to address sex-related differences in terms of therapeutic response and prognosis in HF patients (29). Of note, important sex differences exist in epidemiology, pharmacokinetics, pharmacodynamics, and prognosis, leading to differential responses to pharmacological therapies (30, 31). Nonetheless, women remain consistently underrepresented in interventional trials (20%–25% of overall patients), and, therefore, guidelines are predominantly based on male-derived data. Our GENERATOR HF DataMart, consisting currently of 37.7% women, a percentage significantly higher than that of RCTs and in line with other HF registries, can contribute, at least in part, to fill this knowledge gap (29, 32, 33). In this regard, Figure 8 shows a Sankey diagram, grouping HF patients according to their clinical pathways within our institution. This multi-parametric approach, through which we know about each subject's clinical-laboratory data, comorbidities, and all contacts at our institution, both inpatient and outpatient, allow us to create digital representations of patients (i.e., *digital twins*). Therefore, these population data may be used to build and validate statistical and mechanical models, providing valuable information (phenotyping, risk assessment, prediction of disease development) that, in combination with traditional data, aids in the process of clinical decision-making, in support of precision medicine.

Fourth, big data may be particularly useful when the investigated disease is uncommon, such as congenital cardiovascular disease, a domain in which RCTs often fail to provide definitive answers, because of the small number of subjects enrolled and their inherent heterogeneity (34).

However, the most important drawback of registry-based studies is the lack of randomization of interventions, leading to bias, confounding, and, therefore, the impossibility of establishing treatment efficacy. At the same time, significant obstacles are often encountered in implementing conventional RCTs, especially in the domain of HF, as demonstrated by the small portion of completed studies. Many of these difficulties are mitigated by registry-based pragmatic trials, characterized by simplified regulatory procedures (limited monitoring, regulatory and compliance documentation), focus on essential outcome data, single ethical approval, automatic assessment of outcomes, and real-world evidence (4).

### Limitations

Major shortcomings of big data need to be considered. First, large databases and registries may present operator-to-operator variability in data collection, inconsistent use of definitions, low-quality of data, and missing data (35). RWD is often used for purposes different from those for which they were originally collected and thus may lack information for critical endpoints. In our DataMart a significant proportion of patients did not have an assessment of EF and other basic measurements (i.e., arterial blood pressure and heart rate). When considering the EF data, for instance, the percentage of missing data might be considered relevant, but at the same time provided RWD on the use of the echocardiogram in daily clinical practice. By analyzing of the continuous data and performances derived from our DataMart, a remarkable improvement in the use of echocardiography in the clinical routine was observed, decreasing significantly the incidence of missing and/or not reported data of this parameter when comparing the first year of observation to the third (80+ % missing rate in 2019, reduced to 43% in 2020 and then down to 22% in 2021). However, it should be always taken in to account that the diagnosis of HF is clinically based, and therefore the precise numerical value of EF is occasionally missing since it does not change the patient's therapeutic management, as recently challenged by several authors (24).

In our DataMart, the data collection process is made automatic with regular human validation, which ensures high-quality data, while minimizing missing data. In addition, discrepancies in medical definitions are greatly reduced by having a standardized language in our institution. Second, some analytical issues, such as the potential over-fitting of prediction models and multiple comparisons, should be addressed with appropriate statistical tools, to minimize the likelihood of false-positive associations (15). Over-fitting is a latent problem in predictive modelling, especially in datasets with a limited number of patients and high missing rates. In GENERATOR HF DataMart, we developed models of re-hospitalization and EF variation, employing cross-validation and regularization techniques. Additionally, GENERATOR HF DataMart updates daily with data, providing the possibility to extensively validate and even adapt the developed models. Third, data analysts also encounter considerable difficulties in interpreting the processes by which deep learning algorithms reach their results, the so-called “black-box criticism” (36). Interpretability may be facilitated by capsule-based networks or approaches that systematically censor inputs to define those that have the greatest influence on outputs. Fourth, we acknowledge that the presence of missing data might affect the consistency of our GENERATOR HF DataMart. However, this issue is probably one of the most commonly faced in data cleansing or pre-processing. The degree of missing data will be assessed periodically to quantify the consistency and accuracy of our GENERATOR HF DataMart and will be considered an important quality indicator. By design, regardless of all other factors constituting the data analysis model, the degree of missing data will be minimized as much as possible through the optimization of the processes of data mining and multiple imputations and, in selected cases, by implementing a model to predict the target variable and missing values.

Finally, there are some privacy and bioethical issues due to the pervasive and ubiquitous nature of big data. In this regard, GENERATOR HF DataMart has developed pseudonymization procedures that convert patient-sensitive information into encrypted data, to ensure the usability of potentially sensitive and personal data, while preserving their scalability and reliability (7).

## Conclusions

GENERATOR HF DataMart has been created to support clinical research in the HF field. This is based on an AI-driven process, that automatically extracts data from various sources and uses them for generating clinical evidence, drawn from the real world. This new approach is paving the way for a revolutionary paradigm shift that may provide a complementary perspective to RCTs, while ensuring, at the same time, sustainability, accuracy, velocity, and reproducibility.

## Data Availability

The raw data supporting the conclusions of this article will be made available by the authors, without undue reservation.
