# Supplementary material for: GENERATOR HEART FAILURE DataMart: An integrated framework for heart failure research
Source: Front Cardiovasc Med. 2023 Mar 22;10:1104699. doi: 10.3389/fcvm.2023.1104699 (PMC10073733; doi:10.3389/fcvm.2023.1104699)
Supplement: Supplementary file 1 [file Datasheet1.docx]

**GENERATOR Heart Failure DataMart: an integrated framework for heart failure research**

**SUPPLEMENTARY MATERIAL**

**Supplementary Table 1.** GENERATOR HF DataMart working group.

| **Professional group** | **Professionals** | **Knowledge exchange and decision making** |
| --- | --- | --- |
| Clinical Team | 4 Cardiologists | - Clinical insight on inclusion and exclusion criteria as well as relevant variables to be included in the DataMart during regular working sessions with the technical team. - Contribution to data validation such as medications, comorbidities and risk factors. |
| Technical Team | 2 Data scientists | - Requirements collection and active participation in the implemention of the data extraction, trasformation and validation pipelines. - Coordination of interactive sessions with clinicians and data analysts. - General overview and monitoring of the entire workflow. |
|  | 1 Data analyst | - Data discovery and implementation of data extraction pipelines. - Close collaboration with both clinicians and data scientists during cohort identification, data definition and data validation. |

**Supplementary Table 2.** Data ontology: Variables included in the HF DataMart

| Category | Variable name | Time reference | Type |
| --- | --- | --- | --- |
| Demographics | Age; Gender; Patient ID; Civil status; Educational level | At admission | Structured |
| Admissions | Patient ID; Admission ID; Reason of admission; Order of admission; Date/Time of admission; Date/Time of discharge; Diagnosis;  Emergency visit; Days at hospital; ICU; Type of discharge;  Admission ward; Discharge ward | All | Structured |
| Contacts | Patient ID; Contact ID; Emergency visit ID; Date/Time of contact start; Date/Time of contact end; Type of contact | All | Structured |
| Laboratory exams | Uric acid; Cholesterol; Creatinine; Hemoglobin; White blood cells; Glucose; Lymphocytes; Lymphocytes (%); NT-ProBNP; Potassium; Sodium;  Troponin I Ultra; hs Troponin I  Urea Nitrogen, eGFR | All | Structured |
| Echocardiograms | Ejection Fraction | All | Structured |
| ECGs | R Peak; P Peak; P Duration; PR Duration; QRS Duration;; QTc duration; P Axis; QRS Axis; T Axis; Frequency; Rhythm. | All | Structured |
| Observations | BSA Mosteller;  Body Mass Index; Body Surface Area; Diastolic Blood Pressure; Height; Mean Arterial Pressure; Oxygen Saturation; Pulse; Respirations  Systolic Blood Pressure; Temperature; Weight; | All | Structured |
| Comorbidities | Myocardial infarction; Cardiomyopathy; Arrhythmia  Atrial Fibrillation;  Cardiovascular Disease;  Autoimmune Disease;  Pulmonary Disease;  Neurological Damage;  Arteriopathy; Tumor; Connectivitis;  Renal dysfunction; Hemiplegia;  Dementia; Metastasis; Peptic Ulcer;  Hepatitis; Cirrhosis; Pancreatitis;  Cerebrovascular disease;  Lymphoma; HIV; Hematological Cancer; Stroke; Anemia; Hepatic Disease;  Dyslipidemia; Hypertension;  Diabetes; Hypercholesterolemia | All | Unstructured Clinical history, discharge letters, clinical diaries |
| Risk factors | Smoking; Obesity |  |  |
| Medications | Diuretics; Statins, ACEi; ARNi; MRA; SGLT2i; ARB; β-blockers; Digoxin; Acetylsalicylic acid | At discharge | Unstructured – discharge letters |
| Interventions | Transplant; Chemotherapy; Radiotherapy; Implants; Dialysis |  |  |
| Outcomes | Re-hospitalization; In-hospital Death; Ejection Fraction variation |  | Structured |

**Abbreviations.** ACEi, angiotensin-converting enzyme inhibitors; ARB, angiotensin receptor blockers; ARNi, angiotensin receptor–neprilysin inhibitor; MRA, mineralocorticoid receptor antagonists; SGLT2i, Sodium-glucose co-transporter 2 imhibitors; NYHA, New York Heart Association; ICU, Intensive Care Unit; eGFR, estimated glomerular filtration rate; NT-proBNP, N-terminal pro hormone brain natriuretic peptide

**Supplementary Table 3.** Formulas used for the extraction of calculated variables

| Variable name | Formula |
| --- | --- |
| Estimated glomerular filtration rate (eGFR)[2] | $eGFR_{Cr}=142*{\min\left( \frac{S_{Cr}}{k},1 \right)}^{\alpha}*{\min\left( \frac{S_{Cr}}{k},1 \right)}^{-1.200}*{0.9938}^{Age}*1.012 (if female)$  where:  $S_{Cr}= standardized serum creatinine in mg/dL$  $k=0.7 \left( females \right) or 0.9 \left( males \right)$  $\alpha= - 0.241 \left( female \right) or-0.302 (male)$  $\min\left( \frac{S_{Cr}}{k},1 \right)is the minimum of\frac{S_{Cr}}{k} and 1.0$  $\max\left( \frac{S_{Cr}}{k},1 \right)is the maximum of\frac{S_{Cr}}{k}and 1.0$  $Age (years)$ |
| Body Mass Index (BMI) | $BMI (\frac{kg}{m^{2}})=\frac{weight\left( kg \right)}{\left( height\left( m \right) \right)^{2}}$ |

**Supplementary Figure 1.** Data extraction workflow.


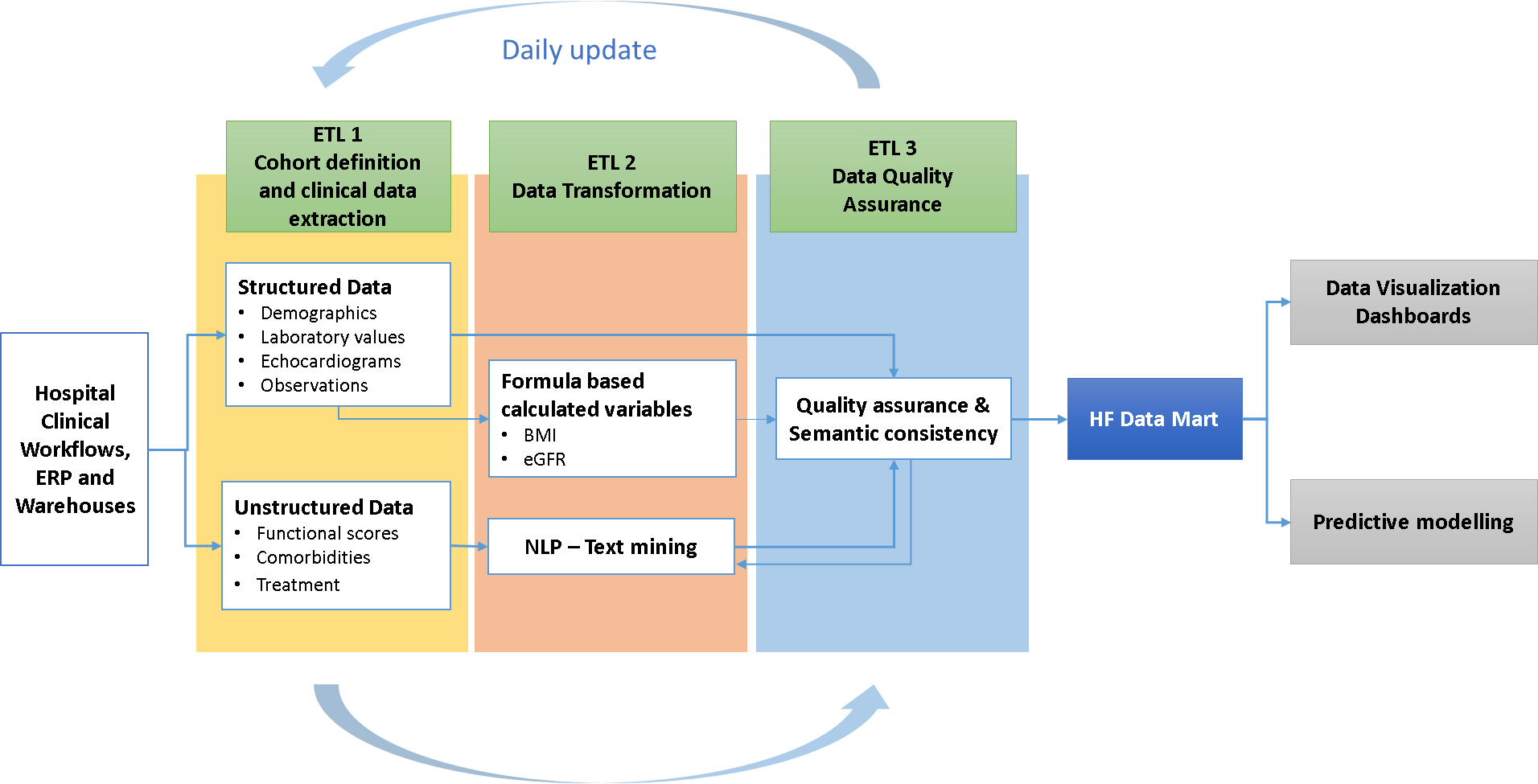


**Abbreviations.** ERP, Enterprise resource planning; ETL, Extract Transform Load; BMI, Body Mass Index; NYHA, New York Heart Association; NLP, Natural Language Processing; eGFR, estimated glomerular filtration rate; HF, Heart Failure

#

**Supplementary Figure 2.** Data model variables mapped to structured and unstructured data sources.


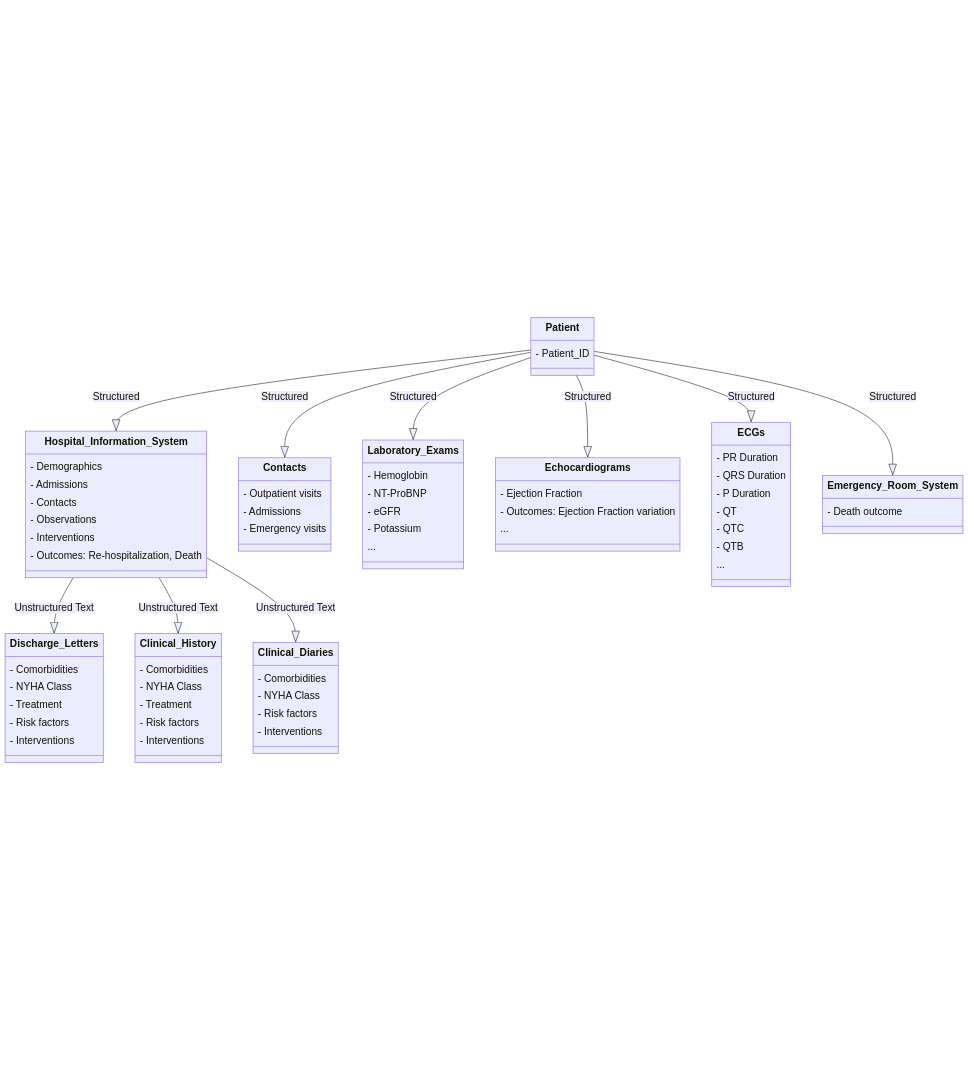


**Abbreviations.** NYHA, New York Heart Association; eGFR, estimated glomerular filtration rate; NT-ProBNP, N-terminal pro hormone brain natriuretic peptide; ECG, Electrocardiograms

#

**APPENDIX**

# **Gemelli Generator DataMart framework**

Heart Failure DataMart's scaffold is based on that of a larger framework previously developed by Damiani et al. (7) at Policlinico Universitario Agostino Gemelli, Rome, Italy.

The implementation of the data science facility addresses three areas:

- *data integration and transformation*:  how efficient extraction and transformation process have been put in place to cover a wide set of healthcare system sources and clinical variables; quality check and validation procedures; user oriented descriptive tools and analytics; statistical analysis and machine learning tools.
- *skill and organization*: how the hospital have been able to establish a multi-disciplinary, collaborative environment where technical staff interacts with non-technical users (clinical staff and medical researchers) to answer scientific research questions through fast data integration, analytics and advanced modelling; what is the process to apply for a new research; how feasibility analysis is performed; timeline for full research execution.
- *ethical and privacy focus*: which data protection and privacy measures are in place, how Data Protection Impact Assessment (DPIA) is performed and which is the collaborative approach with Data Protection Officer to ensure full compliance with General Data Protection Rules; how the laboratory has implemented a standard approach to study protocols in alignment with hospital's Ethical Committee.

DataMart framework is, therefore, based on:

- Gemelli Generator infrastructure, founded on three layers: the data warehouse layers (data lakes of all medical data sources); the datamarts’ layer (first and foremost Generator Data Product, integrated, patient-centered longitudinal history for each pathology): data analytics layer which includes visualization, descriptive tools, statistical and machine learning tools, federated tools.
- Standardized data collection process, which covers an extensive software library for ETL, procedures for data transformation of free-text medical reports (based on Natural Language Processing), specialized search procedures (BOT engines) for diagnoses, procedures, laboratory data, signs and symptoms, comorbidities, and outcomes.
- Modelling tools and use cases which exploit tools addressing: predictive models, risk scores and decision support; patient identification and selection for trials; patients' clustering and digital avatars; conformance checking procedures; radiomics.

The main aim of this framework is to design and implement ‘Gemelli Generator Real World Data’ center in order to create a shared service center where the same approach can be applied to a variety of disease and research questions. Presently, the center has an average throughput of 30 projects in flight, with very high degree of reusability of techniques, consistently with the 'layered' approach described above.

**Gemelli Generator Real World Data ethics and governance**

All RWD studies have research purposes and, therefore, consent-related procedures are consistent with European Directive 2016/679: for all historical patients’ data which are already stored in Gemelli Data Warehouse, no additional consent will be required; for all studies involving new patients and data, informed consent is requested for all patients who are part of the cohorts. Moreover, Gemelli Generator Real World Data aims to accomplish the algorithmic framework of the recent Rome Call for AI Ethic (Rome 26 Febbraio 2020, Pontificia accademia della vita):

1. Transparency: in principle, AI systems must be explainable;

2. Inclusion: the needs of all human beings must be taken into consideration so that everyone can benefit, and all individuals can be offered the best possible conditions to express himself or herself and develop;

3. Responsibility: those who design and deploy the use of AI must proceed with responsibility and transparency;

4. Impartiality: do not create or act according to bias, thus safeguarding fairness and human dignity;

5. Reliability: AI systems must be able to work reliably;

6. Security and privacy: AI systems must work securely and respect the privacy of users.

# **Overview of data extraction procedures**

***Data extraction workflow***

Gemelli Hospital data warehouse integrates several data sources varying from laboratory exams databases, echocardiogram, and electrocardiogram databases to hospital management systems. In GENERATOR HF DataMart, we integrated various data sources implementing dedicated extract, transform and load (ETL) procedures. Each ETL procedure includes data cleaning and data standardization steps to guarantee data compliance to the Data Ontology of reference (Supplementary Table 1). It is worth noting that we used adetailed Data Model based on the Data Ontology presented in Supplementary Table 1 in order to capture the format and the acceptable values for each variable, during ETL procedures implementation.

In technical terms, the GENERATOR HF DataMart is a “relational database” with primary key the patient identifier (patient ID). Such configuration allows for the development of visualization dashboards and models that rely on rich and longitudinal data across different contacts, therefore providing a more comprehensive and intuitive view of disease progression and management.

In Supplementary Figure 1, we summarize the complete data extraction workflow identifying two distinct pathways:

1) ETL 1 captures the cohort definition and the initial data extraction including both structured data and unstructured text data. Regarding structured data, an identification code is associated with each field. The codes used are referred to national and international standards such as The international Classification of Disease (ICD) version 9 ICD9, Diagnosis Related Group Classification and Anatomical Therapeutic Chemical (ATC) Drug Classification. Where none of these standards is available, specific coding for hospital legacy applications is used;

2) ETL 2 instead refers to an additional transformation step that is applied to both structured and unstructured text data. Regarding unstructured text data, the transformation is performed through the application of text mining procedures such as sentences/words tokenization; rule-based algorithms developed in collaboration with the clinicians. In addition, Natural Language processing (NLP) algorithms are employed for data cleaning and standardization such as lemmatization and selection of specific parts of speech. On the other side, selected structured data is used for the calculation of additional variables based on validated formulas such as estimated glomerular filtration rate (eGFR). In Supplementary Table 3, we provide the list of formulas used for specific calculated variables included in the GENERATOR HF DataMart

Finally, ETL 3 implements the data quality assurance process of both structured and unstructured data in a systematic way. With respect to unstructured data, clinicians and IT specialists validate manually a sample of data extracted from text in terms of semantic coherence. The annotations produced are further used for the definition and the improvement of the extraction rules in an iterative fashion.

Based on the data extraction scheme previously detailed, we map all data sources integrated to the GENERATOR HF DataMart (Supplementary Figure 2). Every system presents its own data structure and its integration to the DataMart is performed with the adaptation of the ETL procedures to account for each data structure in an incremental manner.

Finally, the framework presented respects privacy by design by implementing dedicated pseudonymization procedures. Sensitive patient data (such as patient ID and admission ID) is encrypted with the application of the cryptographic hash function MD5. The conversion table is saved into a dedicated and safe area to account for separation of duty and privacy requirements.

***Data quality and completeness***

As explained in previous section, the HF analysis framework includes a dedicated data quality assurance process during data integration in the GENERATOR HF DataMart.

In broad terms, we adopt a validation scheme using evidence from different data sources with the guidance of clinicians, who also conduct independent incremental checks on the quality of the results.

As an example, the assessment of comorbidities could be considered. First, to exploit information from unstructured data in several medical records (such as clinical diaries, nurse diaries, consultancies, diagnostic exams) the clinical team supports data scientists with an annotation phase on a large set of sample documents. The clinical team provides keywords, representative example sentences, indirect evidence included in the documents, which are used by the technical team to implement machine-learning algorithms, i.e. NLP, in order to identify the presence/absence of such comorbidity in the clinical history of a specific patient. Such methods does not imply just the parsing of specific sentences, since the algorithms help identifying semantically equivalent sentences associated to the variable (comorbidity in this example), through the so called 'topic models', and supports the elimination of confounding factors, such as occurrence of negations, reference to familiarities or risk factors..

Once the data scientists have generated a first round of NLP-based identification of comorbidities, a new sample set is used to test the procedure. These new cases are shared with the clinical team in order to perform an independent check, which allows to identify any potential defects generated by the machine learning model, through which the technical team can improve the accuracy of such algorithms.

Then the cross-check validation is performed, which makes use of independent data; for most cases, the occurrence of a comorbidity is associated with well-defined critical ranges for specific laboratory values (such as: glycated hemoglobin, creatinine). This validation, performed on a significant set of test cases, allows to further improve the identification method and adjudicate comorbidities with a high degree of accuracy.

Once the HF Data Mart is built based on the above steps, clinicians have the possibility to control overall consistency with aggregated type of analysis, by using the dashboard linked to the datamart, where they can select subgroups of patients (e.g., the ones characterized with a specific comorbidity) and analyze the distribution of relevant clinical data for such populations.

Finally, as a technical step to evaluate the data quality of the extracted data, we sample several subsets of data (both in patients and variable sets) and we calculate quantitative variable distributions and binary variable incidences among samples. Then, we perform statistical tests to ensure low statistical differences among them and the overall consistency of the entire dataset.
